# The costs of reproduction can and do differ between the sexes

**DOI:** 10.1093/aob/mcaf073

**Published:** 2025-04-21

**Authors:** John R Pannell

**Affiliations:** Department of Ecology and Evolution, University of Lausanne, 1015 Lausanne, Switzerland

**Keywords:** Dioecy, hermaphroditism, trade-off, sexual dimorphism, life-history, sex ratio

## Abstract

**Background:**

Measuring costs of male versus female reproduction in cosexual species is challenging because the currency and timing of allocation can differ between the two sexual functions. In contrast, costs of male versus female reproduction can be measured indirectly in dioecious species in terms of sex-specific life-history trade-offs with growth and survival. Yet despite abundant evidence for differences in life history between males and females, there remains confusion over how such differences should be interpreted.

**Scope:**

Here, I address misconceptions in interpreting potential differences in the costs of reproduction between the sexes, drawing attention to the relevance of: (1) theories of sex-allocation versus life-history evolution; and (2) observations of sex-ratio variation.

**Key Results:**

Sex-allocation theory predicts a mother’s investment in sons versus daughters and is thus relevant to primary sex ratios at the seed stage. Life-history theory is relevant to trade-offs between, for example, reproduction and survival, and is thus relevant to secondary sex ratios of adults affected by sex-biased mortality. The preponderance of species with male- in comparison to female-biased secondary sex ratios points to a frequently greater cost of reproduction for females.

**Conclusions:**

Male and female costs of reproduction often differ, but there remain unanswered questions about why one sex (most often the female function) should often be more expensive than the other. A correct understanding of theoretical predictions will help future research to address such questions.

## INTRODUCTION

It has long been observed that the males and females of dioecious plants frequently differ in traits beyond those that define their sex (Darwin, 1877; Lloyd and Webb, 1977; Geber *et al.*, 1999; Barrett and Hough, 2013). Indeed, although such secondary sexual dimorphism tends to be less striking in plants than it is in many animals (Lloyd and Webb, 1977), very few dioecious plants fail to yield evidence for it when assessed carefully. As first reviewed extensively by Lloyd and Webb (1977) and ~20 years later in a series of chapters in a book dedicated to the subject edited by Geber *et al.* (1999), sexual dimorphism in plants has been documented for a wide range of traits, including those pertaining to the plant life history (reviewed by Delph, 1999; Barrett and Hough, 2013). Life-history differences between males and females include the allocation of resources to flowering and fruiting, with potential implications for differential costs of reproduction as a result of allocation trade-offs (Darwin, 1877; Lloyd and Webb, 1977; Charlesworth and Morgan, 1991; Delph, 1999; Geber *et al.*, 1999; Obeso, 2002; dos Santos *et al.*, 2024). Recently, however, Midgley (2022) has made the bold claim that ‘[t]he costs of reproduction in plants cannot differ between the sexes’, and his paper has begun to be cited as a potentially legitimate view on the subject (e.g. Leal *et al.*, 2023; Scheuerell and LeRoy, 2023).

My purpose in this Perspective is to explain why Midgley’s (2022) arguments are flawed. There are important challenges in understanding costs of reproduction in plants, not least in dioecious or sub-dioecious species that offer fertile ground for within-species comparisons, and further research should be based on sound theoretical reasoning (Charlesworth and Morgan, 1991). Midgley’s (2022) paper makes general assertions about sexual differences and sex ratios of dioecious plants, repeating arguments made previously in the narrower context of the costs of reproduction in males and females of the dioecious Cape genera *Leucadendron* (Midgley *et al.*, 2019) and *Aulax* (Midgley and Cramer, 2022). These papers specifically claim that we should always expect equal allocation to reproduction for males and females of dioecious species, and they cite empirical literature putatively supporting this expectation, with particular emphasis on examples from the Cape of South Africa.

First, I explain why the inference that males and females should be burdened by the same cost of reproduction is based on a misunderstanding of the predictions of sex-allocation theory. Essentially, the inference conflates the theory of sex allocation with predictions from the theory of life-history evolution. Second, I challenge Midgley’s (2022) interpretation of the empirical evidence relevant to differential costs of reproduction between males and females. Although there is plenty of scope for further research, the evidence for differential costs of reproduction is overwhelming. Third, I comment on observations of dioecious species in the Cape flora, which confirm rather than reject expectations for differential costs of reproduction. I end with a brief discussion on estimating costs of reproduction in hermaphrodites, because reservations about the feasibility of measuring costs of reproduction apply more to hermaphroditic species than to dioecious ones.

## COSTS OF REPRODUCTION CONCERN LIFE-HISTORY TRADE-OFFS, NOT SEX ALLOCATION

Midgley’s (2022) conceptual argument that there can be no differences in the costs of reproduction between males and females is erroneous because it muddles predictions of the theory of life-history evolution and sexual selection with the theory of sex allocation. His claim is ‘that theory predicts equal sexual allocation to reproduction’, citing Hamilton (1967) and giving Düsing’s (1884) and Fisher’s (1930) explanation for equal sex ratios in outcrossing populations: in Midgley’s (2022) words, ‘that sex ratios for dioecious organisms must be 1:1 because of the relatively superior fitness of mothers of the rarer sex’. Because every seed must have both a mother and a father, frequency-dependent selection will return sex-biased populations to sex-ratio equality, because parents belonging to the rarer sex necessarily transmit more gene copies to the next generation than those in the majority. Hamilton (1967) generalized Düsing’s (1884) and Fisher’s (1930) explanation by demonstrating how relatedness between the two parents can also account for sex-ratio biases. Predictions for equal and biased sex ratios are supported by a large number of studies across a wide range of taxa and thus represent one of the great successes of evolutionary theory (West *et al.*, 2000; Frank, 2002). In particular, they specifically predict 1:1 primary sex ratios in dioecious plants. Midgley (2022) explains that ‘because males and females typically contribute equally to the genetic composition of the offspring and sex ratios are expected to be equal, the logical expectation is that the sexes allocate equally to reproduction’. But this is false.

The theory of sex allocation, as applied to dioecious species, predicts the ratio of resources allocated by mothers to male versus female progeny (Hamilton, 1967), not the reproductive behaviour of those sons and daughters (Fisher, 1930; Charnov, 1982; Frank, 1986; Hardy, 2002; West, 2009). We have very little data on the primary sex ratios of plants (the sex ratio of seeds at the point of their dispersal) (Barrett *et al.*, 2010; Sinclair *et al.*, 2012; Barrett and Hough, 2013; Field *et al.*, 2013), but it is highly likely to be 1:1 in almost all dioecious species, as predicted by the theory (but see Eppley, 2001; George and Karun, 2011; Morgan *et al.*, 2020). Female-biased primary sex ratios have been inferred for a number of plants, but these exceptions are likely to be the result of differential competition between pollen tubes with X versus Y chromosomes (reviewed by Stehlik *et al.*, 2008), meiotic drive or cyto-nuclear interactions (Hrones *et al.*, 2019; Mao *et al.*, 2024), and they are thus not relevant to the theory of sex allocation (reviewed by Field *et al.*, 2013). Almost all sex ratios reported in the literature are based on counts of adult plants, for which biases can usually be attributed to differential mortality or to differential clonal expansion (Putwain and Harper, 1972; Lloyd and Webb, 1977; Allen and Antos, 1993; Delph, 1999; Van Drunen and Dorken, 2012; Field *et al.*, 2013; Timerman and Barrett, 2019; Bürli *et al.*, 2022). Patterns of survivorship, longevity and clonal growth reflect life-history ‘decisions’ and trade-offs (Stearns, 1976, 1992; Charlesworth and Morgan, 1991), and differences in them between the sexes ultimately point to the differential costs of reproduction that Midgely (2022) disputes.

## EMPIRICAL EVIDENCE FOR DIFFERENTIAL COSTS FOR REPRODUCTION BETWEEN THE SEXES

Midgley’s (2022) commentary prompts us to ask how investment should or even could be demarcated and sensibly costed. This question touches on several operational issues. Should we, for example, measure male costs in terms of investment in pollen or investment in male flowers and all that goes into them to attract, reward and manipulate pollinators (which contributes to mating success)? And how might we calibrate the costs of reproduction incurred by females on those incurred by males, especially if male flowers versus female flowers plus their resulting fruits draw on different limiting resource currencies? Moreover, how should we account for the possibility that different resources might be limiting at different times during the reproductive season? For instance, how do we account for temporal variation in, say, the cost of carbon that is more valuable (because harder to come by) during periods of low solar irradiance, at low temperature or in periods of drought? Finally, how do we take account of the fact that males and females will tend to express phenotypes optimized to harvest those resources particularly needed for their respective reproductive success, rendering the costs per unit invested sex specific?

One response to these questions might be to cast them aside. In Midgley’s (2022) words, ‘at the most basic level evolution is about fitness, thus the life of all males and females is ultimately equally and totally allocated to reproduction’. Thus, because the whole organism has been selected to maximize its reproductive success, estimates of the costs of reproduction must be meaningless. But this dismissal of costs of reproduction as meaningless amounts to rejecting any attempt to understand the differences in life history between males and females, which have been abundantly well documented.

In their seminal review, Lloyd and Webb (1977) assessed the evidence at the time for secondary sexual differences between males and females of dioecious and sub-dioecious plants, including ‘Growth and vegetative reproduction’, ‘Differential survival and sex ratios’, and ‘Reproductive effort’. As noted above, reviews published since have only confirmed the conclusion that females (especially in perennial plants) very often pay a higher cost of reproduction than males, a view supported by the strong association between the reproductive strategy of females (the relative timing, currency and amount of resource allocated to reproduction) and lower growth rates or survival than those of males (e.g. Delph, 1999; Obeso, 2002; Barrett and Hough, 2013). The fact that natural selection has favoured the evolution of divergence in the costs of reproduction between the sexes is interesting, because its explanation is less trivial than has often been supposed. Why should males so often pay a lower price in terms of growth or survival than females? The various possible alternative explanations advanced by Lloyd and Webb (1977) remain poorly tested.

## COMMENTS ON DIFFERENCES IN ALLOCATION BETWEEN THE SEXES IN THE CAPE FYNBOS

To support the view that males and females cannot differ in their cost of reproduction, Midgley (2022) draws on observations of the Cape fynbos flora, on which he is an expert. He argues that a ‘better direct test for the differences in allocation [between the sexes] is whether females in dioecious species produce double the recruits as do co-occurring hermaphrodites’ of other species, with the implication that they could do so only if the reproductive allocation of males, females and hermaphrodites is the same. Of course, co-existence of any two species requires that, over time, their rates of demographic increase are the same (and equal to one). But this places no constraint on their reproductive allocation or effort. Plant communities are almost always maintained with species that have differing life histories (including strategies involving differing emphasis on allocation to reproduction versus growth). If annuals and perennials can co-exist, why should dioecious and hermaphroditic species with different life histories not do so?

Midgley’s (2022) argument here recalls the idea of a ‘seed shadow handicap’, as modelled by Heilbuth *et al.* (2001). Heilbuth *et al.* (2001) showed that females of a dioecious species need to produce more than twice as many seeds as co-occurring hermaphrodites to persist in the same community to overcome the potentially greater competition among progeny clumped around fewer maternal parents, a result that appears to be similar to that invoked by Midgley (2022). However, the model presented by Heilbuth *et al.* (2001) assumes that the two species have the same life history, whereas this need not be so. It is plausible that the seed handicap of dioecious species constitutes a demographic burden not carried by equivalent hermaphrodites, with implications for the persistence and diversification of dioecious lineages, as argued by Heilbuth *et al.* (2001). But this possibility has not prevented the evolution of dioecy from hermaphroditism or the coexistence of dioecious with hermaphroditic species.

Midgley (2022) suspects that the paucity of published estimates of sex ratios for fynbos dioecious plants reflects a negative reporting bias against ‘uninteresting’ 1:1 sex ratios. Field and Barrett (2013) found data on sex ratios from 243 different plant species from 123 genera and 61 families, each species represented by an average of 4.5 populations. For 49.8% of these species, sex ratios did not differ from 1:1, but the other populations were either male biased or, less frequently, female biased. Irrespective of whether fynbos sex ratios are predominantly 1:1 (although see below), the evidence is thus clear that many species do show biases in sex ratios and that many of these biases are attributable to differential mortality or differential clonal growth (Field *et al.*, 2013), as already discussed at length by Lloyd and Webb (1977). Against this background, it is difficult to understand Midgley’s (2022) statement that ‘the evidence that the direct costs of reproduction differ between the sexes is weak and paradoxically papers suggest that there are no indirect [trade-off] costs’. He does not cite Bond and Maze (1999), who specifically reported significantly (and substantially) male-biased sex ratios in all populations surveyed of the dioecious Cape fynbos species *Leucadendron xanthoconus*. Interestingly, Bond and Maze (1999) also reported greater mortality for males with higher reproductive investment. Indeed, their statement that ‘females predominated among live plants in the old dense stand because males had suffered higher mortality’ represents one of the few studies that has made a direct link between reproductive investment and mortality. For a more recent study, in which greater mortality is linked to female reproduction in a North American maple species, see Blake-Mahmud and Struwe (2019). It would be valuable to survey sex-ratio variation in more Cape fynbos species, but a finding of 1:1 sex ratios could hardly overturn the abundant evidence we have from plants for differential costs of reproduction between the sexes of dioecious plants, both in general and, indeed, from the fynbos.

## ‘DIRECT’ VERSUS ‘INDIRECT’ COSTS OF REPRODUCTION IN DIOECIOUS AND HERMAPHRODITIC SPECIES

Midgley (2022) distinguished between ‘direct’ and ‘indirect’ estimates of the costs of reproduction. Direct estimates involve absolute measures of allocation of particular currencies, e.g. biomass, to reproduction by males and females, and Midgley (2022) noted that it is not straightforward to interpret the allocation of a particular currency to male versus female functions in terms of relative costs, a point also raised previously by others (e.g. Antos and Allen, 1990; Charlesworth and Morgan, 1991; Ashman, 1994; Harris and Pannell, 2008). This is because plants may allocate different currencies to male and female functions, potentially committed at different times. For instance, females may allocate more biomass to reproduction than males, but they may do so by being better equipped to harvest carbon, or they may allocate their carbon at a time when its fixation by photosynthesis is easier. It is not uninteresting to find that females allocate more biomass, say, to reproduction, than do males, but Midgley (2022) is right in arguing that measurement of the absolute allocation of a particular resource currency to a particular function tells us little about relative costs of reproduction.

Indirect costs refer to trade-offs between one life-history trait and another, and as such they of course do provide a measure of relative costs, by definition. A female that invests heavily in reproduction might pay the price of reduced survivorship or growth, and the differences in survivorship or growth between males and females are precisely those that measure the costs of reproduction (Lloyd and Webb, 1977; Delph, 1999; Leigh *et al.*, 2006). It is because male and female functions are supported by different individuals for which we can measure differential survivorship or growth, for example, that measuring costs or reproduction is feasible in dioecious populations. But it is more difficult to estimate the relative costs of reproduction through male versus female functions in hermaphrodite populations, especially if all individuals adopt a similar sex-allocation strategy (Charlesworth and Morgan, 1991). Nevertheless, in populations that are canalized for sex allocation, measurement of indirect trade-off costs can be achieved, in principle, by experimental manipulation of investment in one or other of the sexual functions, e.g. preventing allocation to flowers and fruits by bud removal (e.g. Agren, 1988; Charlesworth and Morgan, 1991; Hartemink *et al.*, 2004; Harris and Pannell, 2008; Alvarez-Cansino *et al.*, 2010; Teitel *et al.*, 2016; Rabska *et al.*, 2022).

## CONCLUSION

In conclusion, the claim that costs of reproduction cannot differ between the sexes is incorrect on both theoretical and empirical grounds. The argument falsely appeals to the theory of sex allocation, which is irrelevant to the life-history question of how much individuals should invest in reproduction versus growth and survival. Empirically, it has long been known that many dioecious species are sexually dimorphic for life-history traits, including reproductive allocation and survivorship, and there are many examples of biased secondary sex ratios consistent with differential costs of reproduction, including from dioecious species in the Cape fynbos. Evidence along these lines was reviewed and discussed nearly 50 years ago by Lloyd and Webb (1977), and a great deal more evidence for differential costs of reproduction has accumulated in the interim.

The documentation of yet further evidence relevant to differential costs of reproduction would be valuable, especially when nuanced by analysis of when differences occur and when they do not. It also seems timely to ask why, for instance, the males and females of many species allocate so similarly to reproduction, despite the potential for greater divergence between them, or why, in those species that are dimorphic for life-history traits, females should so frequently pay a higher price of reproduction than males. As was already pointed out by Lloyd and Webb (1977), this fact cannot be attributed to the greater size of ovules and seeds than pollen grains or to the fact that only females produce fruits.
